# Muscle Regeneration and RNA: New Perspectives for Ancient Molecules

**DOI:** 10.3390/cells10102512

**Published:** 2021-09-23

**Authors:** Giulia Buonaiuto, Fabio Desideri, Valeria Taliani, Monica Ballarino

**Affiliations:** 1 Department of Biology and Biotechnologies “Charles Darwin”, Sapienza University of Rome, 00185 Rome, Italy; giulia.buonaiuto@uniroma1.it (G.B.); fabio.desideri@uniroma1.it (F.D.); taliani.1690724@studenti.uniroma1.it (V.T.); 2 Center for Life Nano & Neuro-Science of Instituto Italiano di Tecnologia (IIT), 00161 Rome, Italy

**Keywords:** noncoding RNAs (ncRNAs), RNA therapeutics, skeletal muscle regeneration, cardiac regeneration

## Abstract

The ability of the ribonucleic acid (RNA) to self-replicate, combined with a unique cocktail of chemical properties, suggested the existence of an RNA world at the origin of life. Nowadays, this hypothesis is supported by innovative high-throughput and biochemical approaches, which definitively revealed the essential contribution of RNA-mediated mechanisms to the regulation of fundamental processes of life. With the recent development of SARS-CoV-2 mRNA-based vaccines, the potential of RNA as a therapeutic tool has received public attention. Due to its intrinsic single-stranded nature and the ease with which it is synthesized in vitro, RNA indeed represents the most suitable tool for the development of drugs encompassing every type of human pathology. The maximum effectiveness and biochemical versatility is achieved in the guise of non-coding RNAs (ncRNAs), which are emerging as multifaceted regulators of tissue specification and homeostasis. Here, we report examples of coding and ncRNAs involved in muscle regeneration and discuss their potential as therapeutic tools. Small ncRNAs, such as miRNA and siRNA, have been successfully applied in the treatment of several diseases. The use of longer molecules, such as lncRNA and circRNA, is less advanced. However, based on the peculiar properties discussed below, they represent an innovative pool of RNA biomarkers and possible targets of clinical value.

## 1. Introduction

*Freewheeling*, *audacious,* and *non-conformist*: These terms perfectly define the unique nature of RNA and underline the differences with DNA, *straightlaced* and *uniform* by virtue of its genetic responsibilities [1]. In 2020, two major events raised RNA to prominence. In the genetic engineering field, it is notable to mention the Nobel Prize in Chemistry awarded for the development of the CRISPR/Cas9 technology [2,3]. Here, the presence of an RNA guide within the proteinogenic Cas9 complex ensures accuracy and makes these genetic scissors excellent candidates for pharmaceutical applications and precision medicine [4]. A further contribution was made in relation to the COVID-19 pandemic, which engaged academia and biotech in an unprecedented effort to find rapid solutions to contain virus spread. Decades of research on RNA vectors [5] and RNA-based vaccines [6] designated the ribonucleic acid as safe and effective to develop vaccines for humans. By December 2020, two mRNA-based vaccines (Moderna and Pfizer/BioNTech) completed the clinical trials, started to be administrated at a large scale [7], and gave successful performances in producing high and persisting titers of antibodies [8]. As rarely done before, the need to produce new knowledge on RNA opened new frontiers of research, which is expected to lead to original solutions for therapies and biopharmaceuticals.

The existence of distinct roles for RNA was initially suggested by the discovery of messenger (mRNA) [9,10], ribosomal (rRNA) [11,12], and transfer (tRNA) [13] RNAs. Later on, several classes of relatively small non-coding (nc)RNAs were also identified, such as the small nuclear (snRNA) [14,15], small nucleolar (snoRNA) [16,17], micro (miRNA) [18], piwi-interacting (piRNA) [19], and small interfering (siRNA) [20,21] RNAs. Among them, miRNAs have attracted considerable attention because of their participation in almost every aspect of physiological [22,23] and pathological [24,25,26,27,28] processes. 

In the last years, research on RNA was fostered by the emergence of the Next-Generation Sequencing (NGS) technologies, which offered the chance to deepen the analysis of multiple (cell and tissue) transcriptomic landscapes [29,30,31,32,33]. As reported in the latest Ensembl release [34], in humans, this yielded a number of ncRNAs significantly higher than the coding ones (23,982 versus 20,442) and mainly represented (~16,896) by long non-coding RNAs (lncRNA). LncRNA constitute the most recent and heterogeneous class of ncRNAs acting at transcriptional as well as at post-transcriptional levels through a variety of mechanisms [35,36,37]. A distinctive class of lncRNAs is constituted by the circular (circ)RNAs, whose covalently closed structure is key to their exceptional stability in the cellular environment [38,39]. As such, they have evolved conserved roles in multiple physiological processes and their involvement in pathology has deserved increasing consideration from the scientific community [40,41,42].

Herein, we will highlight the most recent examples of RNAs implicated in the pathophysiology of muscle, with a glance at their use in clinical trials. While the literature on RNA and myogenesis is vast, we will specifically focus on the importance of coding and non-coding RNAs to the process of regeneration for both, skeletal and cardiac muscles. Current analyses in this field still pose unanswered questions as to whether a stem cell compartment in the adult muscle exists, how long it maintains its regenerative potential, or how it can be regulated [43]. In skeletal muscle, regeneration is driven by a reservoir of resident progenitors, called satellite cells, able to efficiently replenish damaged muscle [44]. These cells are not present in the adult cardiac muscle, although a regenerative response, mediated by the proliferation of pre-existing cardiomyocytes, occurs in mice during the first week of life [45,46,47]. Temporal and tissue-specific nuances in the process of regeneration may underlie the participation of still unknown protagonists, whose ability to fine-tune myogenic expression becomes critical in both physiological and pathological conditions. The peculiar properties of RNA, along with its tissue specificity, satisfy the requirements for its integration in regenerative networks and will surely pave the way for future applications in medicine.

## 2. Chemical and Structural Properties Define Multiple Arrays of RNA Functions

For a very long time, researchers have documented only one of the RNA-specific tasks, which was related to its ability to code. Later studies have revealed that, instead, RNA can play additional functions and act as a non-protein-coding protagonist [48]. As such, its chemical character as a single-strand polymer becomes fully exploited, enlarging the physiological schemes of regulation and the development of new therapeutic tools.

**Figure 1:**
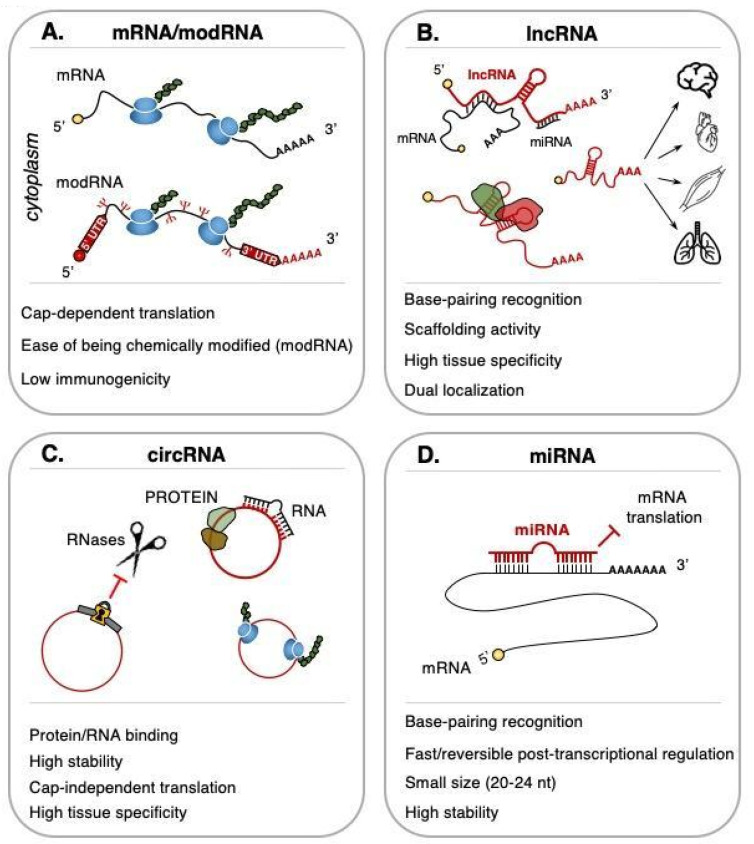
Chemical and structural properties of RNA that are usable for therapeutics. Different RNA types are shown as follow: (**A**) mRNA/modRNA. The uridine into pseudouridine substitution in modRNAs is represented by the greek Ψ symbol (red); (**B**) lncRNA; (**C**) circRNA. Locket (yellow) represents the covalently closed structure which makes circRNA resistant to exonucleases (RNases); (**D**) miRNA. See text for further details.

Historically, the potential of being translated upon exogenous delivery has made mRNA the most suitable vehicle for the expression of almost any kind of protein, including antibodies [49,50], antigens [51], or cytokines [52] (Figure 1A, mRNA). The relatively short half-life and the cytoplasmic localization of mRNA bypass genomic integration and mutagenic problems, thus ensuring an high safety profile [53]. Undoubtedly, the intrinsic susceptibility to degradation by nucleases represented a weakness for its systemic delivery. Depending on the target tissue, this led to formulating different strategies to optimize the intracellular intake of the mRNA cargo, which include viral or lipid-based (i.e., lipoplex or lipid nanoparticles) vectors for encapsulation. The addition of chemical modifications at the level of 5′-cap [54], poly(A) tail [51], or at specific nucleotides [55,56] was also instrumental for RNA stabilization and uptake (Figure 1A, modRNA) [57].

The biochemical versatility of RNA is maximized by certain lncRNA features, such as their dual (nuclear and/or cytoplasmic) distribution and the extraordinary cell-type and timely regulated expression [58,59]. Notably, the participation of lncRNAs in specific nuclear processes (Figure 1B) opens up a chance to develop early intervention approaches to treat diseases, especially those characterized by comorbidity or without a clear genetic cause. In muscle, a plethora of different mechanisms executed by nuclear and cytoplasmic lncRNAs have been detailed [60]. These molecules are generally classified based on their length (longer than 200 nt), many of them are transcribed by RNA polymerase II (RNA Pol II), and display features in common with mRNAs, such as the presence of a 5′-cap, a 3′-poly(A) tail, and splicing consensus sequences [61,62]. Alternative splicing and intron retention were also shown to be important for the subcellular distribution and the functions of these RNAs [63,64]. In the nucleus, lncRNAs act as potent epigenetic regulators (i.e., chromatin modifiers or 3D architects); in the cytosol, they influence the mRNA stability by acting as protein or miRNA sponges [65,66]. Stability is the extra value of circRNAs (Figure 1C), for which the lack of free 5′ and 3′-ends prevents degradation by the endogenous RNases [67]. This abundant class of molecules is generated from a non-canonical splicing event, called backsplicing, which occurs through the formation of a covalent bond between a downstream splice-donor site and an upstream splice-acceptor site [39]. Along with the ease of purifying from peripheral fluids [68,69] and the high stability, the altered expression of circRNAs in both muscular [70] and cardiovascular pathologies [71] encouraged their use as non-invasive and quantifiable biomarkers. This application is expected to burst in the next few years to diagnose specific pathological states and monitor therapeutic outcomes.

A different set of features applies to small ncRNAs (of length shorter than 200 nt). MiRNAs (Figure 1D) are transcribed by the RNA Pol II as long primary transcripts (pri-miRNA) and processed in the nucleus by the Microprocessor complex, which contains the RNase III-like enzyme Drosha and its cofactor DGCR8 [72]. Previous studies on Drosha cleavage suggested that the ribonuclease activity is potentially enhanced and contemporary to transcription for both intron-encoded and intergenic miRNAs [73,74]. Upon nuclear processing, the 70 nt-long intermediate named pre-miRNA is exported to cytoplasm and converted into a mature miRNA duplex by the RNase III endonuclease Dicer [75]. This duplex is subsequently loaded onto Argonaute to form the effector RNA-induced silencing complex (RISC). The ability to base-pair with complementary sequences located in the 3′UTR of specific mRNAs represents a major determinant of miRNA regulation and allows their use in therapeutics. In addition, the small (18–24 nt) size supports their stability and is practical for manipulation and delivery, thus making miRNA detection relatively easy in plasma and serum [76,77], saliva [78], and urine [79]. As of now, several studies have reported the validity of using miRNAs as biomarkers for several conditions, ranging from cancer to myocardial infarction [80,81,82].

## 3. Applications of Small and Long RNAs as Therapeutic Tools for Muscle Regeneration

The therapeutic use of different RNA types is not only feasible but also extremely promising. However, despite the overall benefits discussed above, prior publications mostly referred to the small species, such as miRNAs. When it comes to longer RNA molecules, examples of their applications in muscle regeneration are very limited and are mainly represented by the use of mRNA. In fact, contrarily to miRNAs and mRNAs, many lncRNAs are still poorly characterized. The lack of gene annotations and their low abundance required the set-up of orthogonal “omics” to describe the structure, the interactions, and, importantly, to provide evidence on the biological significance of these transcripts [83,84]. Nevertheless, examples of lncRNAs relevant to myogenesis and potentially useful as therapeutic tools are starting to surface and will be presented below.

### 3.1. Small Non-Coding RNAs

#### 3.1.1. microRNA (miRNA)

**Figure 2:**
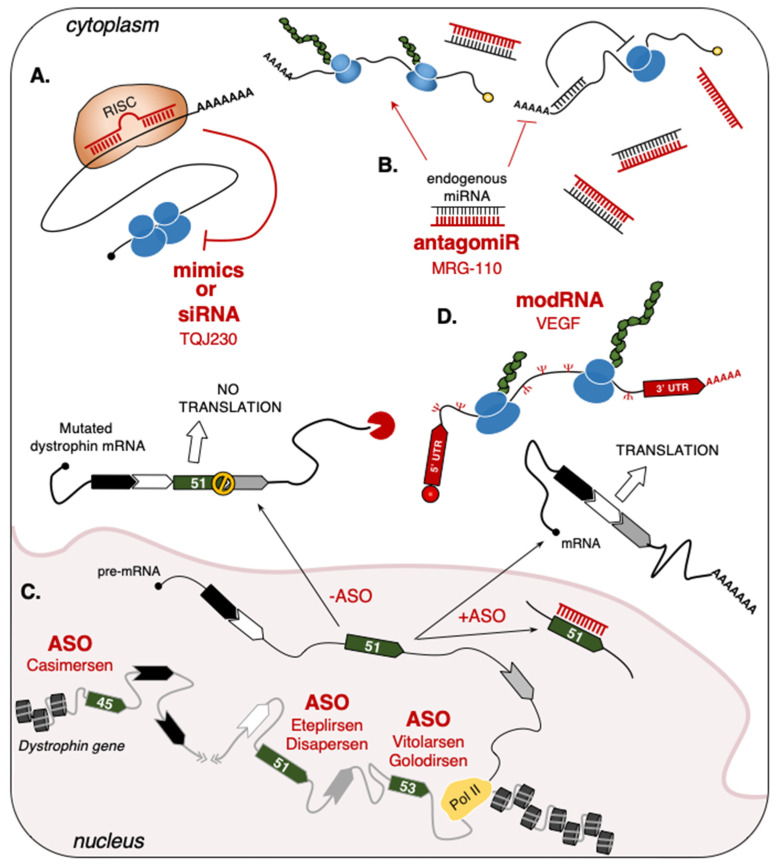
RNA-based drugs in muscular and cardiovascular pathologies. (**A**) “Mimics” and siRNAs act by targeting specific mRNAs to inhibit their translation. Examples include the TQJ230 siRNAs, which specifically recognize and induce the degradation of ApoA mRNA in patients with pre-existing cardiovascular diseases [91]; (**B**) “AntagomiRs” act by sponging endogenous miRNAs, thus preventing their translational repression. MRG-110 was used to block miR-92a activity on pro-angiogenic genes to induce wound healing [92]; (**C**) ASO can be used to modify the splicing of precursor mRNAs (pre-mRNA). The exon-skipping strategy applied to dystrophin exon 51 is shown as an example. In DMD patients (-ASO), genetic mutations lead to the formation of a premature stop codon (STOP symbol) in the mature transcript that causes the lack of protein translation. The use of ASO base-pairing with dystrophin exon 51 (+ASO) promotes its exclusion from the mature mRNA and leads to the translation of a shorter (but functional) protein. For each targeted exon, the ASO approved by the FDA (Food and Drug Administration) are indicated [93,94,95,96,97]; (**D**) VEGF modRNA used in MI patients [98]. The uridine into pseudouridine substitution is represented by the greek Ψ symbol (red). Grey line: DNA; black line: RNA; red line: Therapeutic RNA.

For therapeutic purposes, researchers can use small ncRNA-based drugs functioning as miRNA “mimics” (Figure 2A) or “inhibitors” (antagomiR) (Figure 2B). While “mimics” are designed to imitate [85], “antagomiRs” instead counteract endogenous miRNA activities [86]. These types of drugs have been applied to several diseases, such as blood cancer (antagomiR-155, Cobomarsen) [87], Alport’s nephropathy syndrome (antagomiR-21, Lademirsen) [88], and malignant pleural mesothelioma (mimic_miR-16, TargomiRs) [89] and also represent a prevailing revolution in the field of muscle regeneration, as demonstrated by their use in animal models (i.e., mice, rats, and pigs) [90].

In cardiac muscle, one of the challenges being tackled by researchers using these drugs was to re-establish heart functionality upon myocardial infarction (MI). In particular, different strategies have attempted to revert the necrotic death caused by MI-dependent hypoxia, either by increasing the proliferation of cardiomyocytes or by developing new blood vessels [99]. An example is represented by miR-199-a, a highly conserved miRNA shown to stimulate cardiac regeneration by promoting cell-cycle re-entry of adult rat cardiomyocytes [100]. Similarly, intramyocardial AAV6-injection of miR-199-a mimics in pigs, which underwent infarction by coronary artery occlusion, was found to stimulate cardiomyocytes proliferation [101]. The treatment also ameliorated the overall cardiac conditions by reducing the MI size and fibrosis and by improving the contractile functions. However, the inability to control the number of immature cardiomyocytes led to adverse effects, as 70% of the treated pigs died 7–8 weeks after injection [101]. Another example is miR-325-3p, of which administration in MI mice reduced the myocardiac damage through the repression of the necroptotic factor RIPK3 (Receptor Interacting Protein Kinase 3) [102,103]. Conversely, inhibition of miR-325-3p by antagomiRs produced an increase in apoptotic cardiomyocytes and MI size, which confirms the biological relevance of miR-325-3p in the progression of MI through the RIPK1/RIPK3/p-MLKL axis [103]. In a study published in 2012 [104], Ucar and coworkers assigned pro-hypertrophic functions to the miR-212/132-3p miRNA family. The hypertrophic stimulus was carried out by the miR-mediated regulation of two key genes, the anti-hypertrophic and pro-autophagic FoxO3 transcription factor [105] and SERCA2a, a sarcoplasmic/endoplasmic reticulum ATPase regulating calcium flow during cardiac contraction [106]. Foinquinos and colleagues used two animal models to test the miR-212/132-3p activities on the capacity of the heart to recover after stress, specifically, a transgenic mouse model overexpressing the miR-212/miR-132-3p family and a pig model of heart failure. In both the animals, the systemic administration of miR-132-3p antagomiRs rescued the development of cardiac hypertrophy, reduced left ventricle remodeling, and improved cardiac function [106]. Given these promising results, miR-132-3p antagomiRs (CDR132L) are currently being tested on human heart failure patients. So far, this study has revealed that intravenous infusion of CDR132L is safe and well-tolerated [107]. A phase I clinical trial is currently ongoing to test MRG110, an antagomiR-based drug that inhibits miR-92a in endothelial cells (Table 1). As miR-92a is involved in the repression of pro-angiogenic genes (i.e., the α5/αv integrin subunits and the SIRT1 histone deacetylase) [108], the aim of this strategy is to accelerate wound healing by improving the blood flow within the injured area [90,92]. The use of miR-92a antagomiRs was proven to be successful in cardiac-injured mice [108] and pigs [109] for enhancing angiogenesis and reducing infarct size, with a consequent improvement of overall cardiac functions. Pigs also showed reduced cardiac inflammation, as compared to control animals [109]. This is particularly promising as (i) miR-92a is upregulated upon cardiac ischemic injury in several animal models [108,109] and (ii) vascularization improvement represents the most promising strategy to reduce the effects of MI.

In skeletal muscle, regeneration follows a very different path in respect to the heart due to the presence of satellite cells, the most representative muscle stem cells. Historically, satellite cells’ specification and self-renewal were ascribed to the activity of the paired-box Pax7 transcription factor [110,111,112]. The fact that, in mice, the regulation of Pax7 levels by miR-1/miR-206 influences the commitment of satellite cells from self-renewal to differentiation [112] paved the road for the use of miRNA-based drugs for the treatment of skeletal muscle diseases. In mice, miR-127 regulates the translation of S1PR3 (Sphingosine 1 Phosphate Receptor 3), a protein involved in the maintenance of satellite cells quiescence [113]. Mice engineered to overexpress miR-127 and subjected to skeletal muscle injury by cardiotoxin show increased satellite cells’ differentiation and accelerated regeneration. Interestingly, miR-127 overexpression also produced a beneficial effect in murine dystrophic muscles [114], which suggested potential applications for the treatment of muscular dystrophies. In the same year, Li and colleagues demonstrated the efficacy of miR-29b-based drugs in atrophy. In rodents, miR-29b is upregulated in multiple types of skeletal muscle atrophy models, which parallels with decreased levels of its direct targets, such as IGF-1 (Insulin-like growth factor 1) and PI3K (p85a) (Phosphatidylinositol 3-Kinase 85 KDa Regulatory Subunit Alpha), both involved in the mTOR signaling pathway [115,116]. MiR-29b inhibition through intramuscular antagomiRs injection was sufficient to attenuate atrophy and to increase the gastrocnemius-weight/body-weight ratio and myofibers diameter [117].

#### 3.1.2. Single-Stranded Antisense Oligonucleotides (ASO)

ASO provided good performances in the treatment of several muscle pathologies [118]. They can be divided into two main categories: The DNA-based ASO, which induces target degradation through the recruitment of RNase H1 [119] and the RNA-based ASO, which alters mRNA processing [120] or translation [121,122] by a base-pairing block. In 2016 and 2017, respectively, the FDA (Food and Drug Administration) and EMA (European Medicine Agency) agencies approved Spinraza [123], the first RNA-based ASO found to be effective in the treatment of the spinal muscular atrophy (SMA) [124] (Table 1). In 2016, the FDA also approved Eteplirsen-ASO [125] for use in patients affected by Duchenne Muscular Dystrophy (DMD). This pathology is caused by several types of mutations of the dystrophin gene, which lead to the formation of premature stop-codons in dystrophin mRNA with the consequent loss of protein expression. Over the years, the use of ASO-based drugs able to convert the out-of-frame mutation to in-frame deletions to produce a shorter, but functional, dystrophin protein has been steadily increasing [126]. To date, the exons targeted by this strategy are represented by exon-51 (Eteplirsen, Drisapersen), exon-53 (Vitolarsen, Golodirsen), and exon-45 (Casimersen) (Figure 2C and Table 1) [93,94,95,96,97]. In particular, Eteplirsen is a 30-nucleotide phosphorodiamidate ASO that induces the skipping of dystrophin exon-51 by impeding the recognition of its splicing sites, thus preventing the formation of a premature stop codon [125]. Even though Eteplirsen was proven to be successful, the treatment can only be applied to ~14% of all DMD patients that present this specific type of mutation [127].

While no clinical trial is currently ongoing, promising ASO-based approaches are being applied in mice that model different muscle pathologies, such as centronuclear myopathies [128,129,130] and myotonic dystrophy type 1 (DM1). DM1 is a multisystemic disorder characterized by myotonia, progressive muscle wasting, cardiac conduction defects, and cognitive impairments [131]. It is caused by the abnormal expansion of CTG repeats in the 3′UTR of *DMPK* (dystrophia myotonica protein kinase) transcripts [132] that induces their nuclear retention [133] and sequestration of several RNA-binding proteins, which functional alteration leads to splicing errors [134]. Subcutaneous injection of ASO against DMPK in different DM1 mouse models has yielded positive results in reducing splicing errors, myotonia, and cardiac defects while increasing both skeletal muscle strength [135,136] and the number of satellite cells [137], thus facilitating the regeneration process.

In the future, several efforts will be made to improve the stability and uptake of ASO in vivo [138]. In this direction, the subcutaneous injection of palmitate-conjugated ASO against PLN (phospholamban) was already shown to improve cardiac dysfunction in mouse and rat heart-failure models and increase survival [139]. For Parkinson’s disease, the use of amido-bridged nucleic acids (AmNA)-modified ASO against SNCA (synuclein alpha) facilitated their intracerebroventricular injection, without the need for additional chemicals [140]. From this perspective, this implementation can help to reduce the dose of injected chemicals during clinical treatments, thus minimizing, as much as possible, patients’ negative reactions.

#### 3.1.3. Short-Interfering RNA (siRNA)

Other strategies that employ small RNAs are based on the use of small interfering RNAs (siRNAs), which exploit RISC to base-pair and degrade target mRNAs, thus impeding the production of the corresponding protein [141]. Along the years, the efficacy of these molecules has been tested in clinical trials for muscular as well as non-muscular diseases, ranging from polyneuropathy (Patisiran) [142] and chronic hepatitis B viral infection (1JNJ-3989) [143] to different types of cancer, such as pancreatic cancer (siG12D-LODER) [144] and hepatocellular carcinoma (TKM-080301) [145]. In cardiac muscle, these agents are currently being tested in patients with pre-existing cardiovascular diseases. For instance, the administration of TQJ230 siRNAs is shown to inhibit the production of the Apolipoprotein-a (ApoA) and reduce the inflammatory activity of circulating monocytes (Figure 2A and Table 1) [91,146]. 

### 3.2. Long-Sized RNAs

#### 3.2.1. Protein-Coding RNAs

mRNA is the ideal instrument for treatments that require the expression of specific proteins. Over the years, this opportunity has inspired researchers to find new strategies for increasing its stability and minimizing immunogenicity through the modification of specific nucleosides. This culminated with the production of modRNAs, synthetic and chemically modified mRNAs originally applied in phase I and II clinical trials (https://clinicaltrials.gov accessed on 10 September 2021) to prevent virus infections, such as Coronavirus (NCT04470427), Zika virus (mRNA-1893, NCT04064905; NCT04917861), and Cytomegalovirus (mRNA-1647, NCT04232280), or in the treatment of solid tumors [147]. In cardiac muscle, modRNAs represent a chance for future MI treatments. As for miRNAs, modRNA-based recipes are thought to stimulate cardiomyocytes’ proliferation and increase the blood flow to the wounded area. For example, VEGF-A (Vascular Endothelial Growth Factor-A) is part of a large family of paracrine factors regulating angiogenesis, endothelial cells’ proliferation, and endothelial precursor cells’ differentiation [148]. First tested in cardiac-injured mice [149,150], pigs and monkeys [151], the direct delivery of VEGF-A modRNAs through epicardial injection yielded encouraging results in terms of survival, by increasing the density of capillaries surrounding the heart and by reducing apoptotic and scarred areas (Figure 2D and Table 1). Moreover, clinical testing demonstrated that the intradermic injection of VEGF-A can increase blood flow in the skin surrounding the injected area and is well tolerated by human patients [98]. To note, a phase 2a clinical trial in a cohort of patients undertaking Coronary Artery Bypass Grafting surgery is currently ongoing to test the efficacy of this drug in cardiac recovery [152]. Besides VEGF-A, two other modRNAs have shown encouraging results by preclinical tests in animal models. The use of the human FSTL-1 (Follistatin-related protein 1) modRNA, chemically modified at the level of N-glycosylation sites, was shown to increase the proliferation of neonatal and adult MI cardiomyocytes, without triggering cardiac hypertrophy [153]. A similar cardio-protective environment was created by treatments that use constitutively active YAP (hippo-Yes-Associated Protein) modRNAs, appropriately modified to inhibit YAP phosphorylation by the Hippo kinase. Since the constitutive activation of the YAP cascade is known to increase the oncogenic risk [154], in mice the transient administration of YAP modRNAs after ischemic injury represented one of the safest manner to improve heart function by reducing necrotic cardiomyocytes and the inflammatory responses [155]. 

#### 3.2.2. Non-Coding RNAs

##### Cytoplasmic ncRNAs

**Figure 3:**
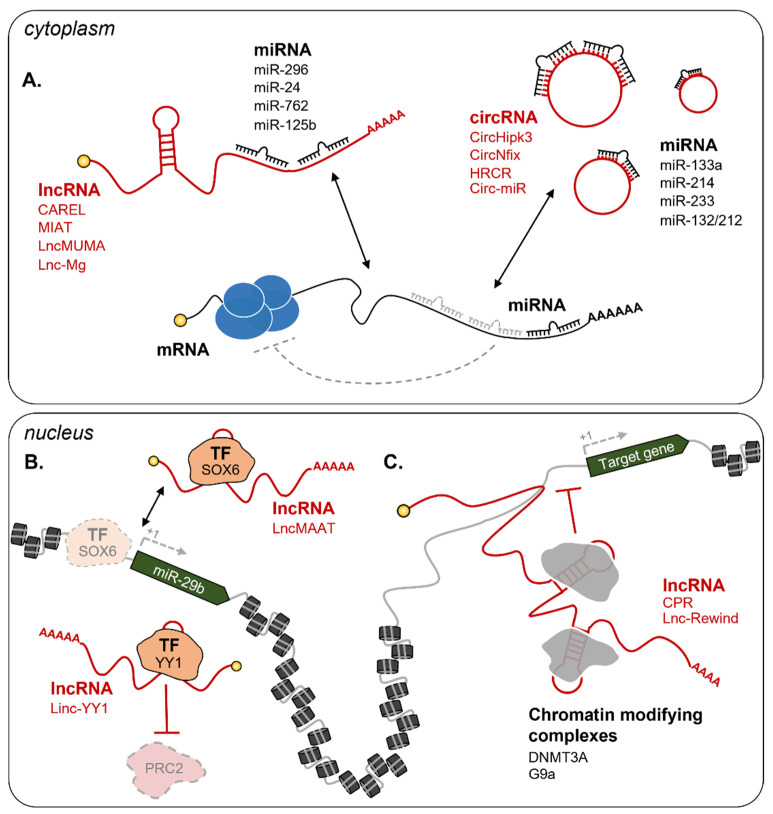
Examples of lncRNA circuitries in muscle regeneration. (**A**) In the cytoplasm, lncRNAs and circRNAs can act as competing endogenous RNAs (ceRNA) to interfere with miRNA binding to their targets. Examples include CAREL/mir-296 [161], MIAT/miR-24 [162], LncMUMA/miR-762 [163], Lnc-Mg/miR-125b [164], CircHipk3/miR-133a [165], CircNfix/miR-214 [166], HRCR/miR-233 [167], and Circ-miR/miR-132/212 [168]. In the nucleus, lncRNAs can influence gene expression at the epigenetic level through several mechanisms [65]. Examples in the figure include (**B**) lncRNA decoys: LncMAAT impedes SOX6 binding on the promoter of miR-29b to repress its transcription [169], Linc-YY1 binds YY1 and blocks its interaction with the PRC2 complex [170]; (**C**) lncRNA guides: CPR [171] and Lnc-Rewind [172] respectively interact with the DNMT3A and G9a repressive complexes and guide them on specific promoters. Dashed grey lines represent the loss of interaction and regulation. TF = Transcription Factor. See text for further details.

The functional participation of lncRNAs in muscle regeneration makes them promising targets for clinical applications. In particular, their ability to act as competing endogenous RNAs (ceRNA) attracted the scientific community and currently represents the most exploited way to dose the relative abundance of miRNAs and their targets in vivo (Figure 3A) [156,157,158]. Starting from 2011, several lncRNAs were shown to act in the cytoplasm of muscle cells as miRNA sponges [65]. One of the first studies led to the identification of linc-MD1, a lncRNA that governs the timing of skeletal muscle differentiation by sponging miR-133 and miR-135 [159]. Upon induction of myoblasts’ differentiation, linc-MD1 starts to be transcribed and inhibits miR-133 and miR-135 activities on their respective targets, MAML1 (Mastermind like transcriptional coactivator 1) and MEF2C (Myocyte enhancer factor 2C). Both proteins are important factors for the transcriptional regulation of pro-differentiating genes [160], thus the ncRNA-mediated regulation of their expression is essential for the correct induction of the latest stages of myogenesis.

Over the years, other lncRNAs were shown to act as ceRNA for miRNAs [173,174]. A recent example is CAREL, a cardiac regeneration-related lncRNA regulating cardiomyocytes proliferation and cardiac regeneration as a miR-296 sponge [161]. Among the known miR-296 targets, the Integral membrane protein 2a (Itm2a) inhibits the growth of breast cancer cells through autophagy induction [175,176], while Trp53inp1 (Tumor protein p53-inducible nuclear protein 1) regulates cell stress responses by inducing cell cycle arrest and apoptosis [177,178]. In neonatal and adult mice subjected to MI, CAREL down-regulation by intramyocardial injection of short hairpin RNAs (shCAREL) enhanced cardiac regeneration by increasing cardiomyocytes mitosis. Mechanistically, this is due to the repression of Itm2a and Trp53inp1 translation promoted by the increased availability of miR-296. Intriguingly, CAREL expression is conserved in human and its depletion in hiPSC-derived cardiomyocytes leads to similar effects [161], opening future investigations into therapy. Another example is MIAT (Myocardial infarction-associated transcript), a lncRNA originally discovered in a large-scale single nucleotide polymorphism association study as linked with an increased MI risk [179]. In cardiac fibroblasts, MIAT sponges miR-24, a miRNA known to attenuate fibrosis in the infarction border zone by inhibiting the translation of Furin, a member of the TGFβ pathway [180]. Accordingly, MIAT downregulation by siMIAT, injected directly into the heart of MI mice, produced a significant reduction of interstitial fibrosis [162]. LncMUMA (Mechanical Unloading-induced Muscle Atrophy) was identified as the most downregulated lncRNA expressed in atrophied skeletal muscles [163]. It represents an extremely promising target since it acts as a sponge for miR-762, a miRNA targeting MyoD, a master regulator of myogenic differentiation. In line with this, transgenic mice expressing high levels of miR-762 in skeletal muscle are characterized by decreased MyoD levels, reduced mass, and small myofibers. MiR-762 repressive effects are rescued by the injection of LncMUMA in gastrocnemius, thus demonstrating the sponging efficacy of the lncRNA. LncMUMA overexpression also has beneficial effects in mice with induced skeletal muscle atrophy through mechanical unloading by the regulation of the same partners [163]. Finally, lnc-Mg promotes MuSC differentiation by increasing Igf2 protein levels through the sponging of miR-125b. Lnc-Mg knock-out mice are characterized by skeletal muscles with an increased number of thinner myofibers that result in reduced muscle weight, strength, and performance. Instead, Lnc-Mg overexpressing mice show increased protection against skeletal muscle mass loss upon denervation, compared to WT animals [164].

Modulation of CircHipk3 expression in MI mice impacts on cardiac regeneration through different targets. In fact, while in cardiomyocytes CircHipk3 influences the acetylation and the half-life of N1ICD (Notch1 intracellular domain) [181], in endothelial cells it acts as a miR-133a sponge to regulate the expression of CTGF (Connective Tissue Growth Factor). Accordingly, CircHipk3 overexpression in MI mice leads to increased cardiomyocytes proliferation with a consequent reduction of the fibrotic area surrounding the infarction zone and the induction of coronary vessel endothelial cell proliferation, migration, and tube-forming capacity [165]. CircNfix is a conserved and cardiomyocyte-enriched circRNA influencing regenerative repair and functional cardiac recovery after MI. CircNfix promotes, on the one hand, the ubiquitination and the consequent degradation of YBX1 (Y-Box Binding Protein 1), a transcriptional repressor of pro-proliferative genes [182]. On the other hand, CircNfix acts as a sponge for miR-214 to promote the expression of Gsk3β (Glycogen synthase kinase 3 beta), a kinase with important roles in both angiogenesis and cardiomyocytes proliferation [183,184]. In line with this, CircNfix knock-down in mice increases angiogenesis in the peri-infarcted area and induces a temporary de-differentiation of cardiomyocytes, with an increment in their proliferation. Together with the heightened proliferation rate, these mice also show a reduction in cardiomyocytes’ apoptosis and fibrosis and improved cardiac function [166]. Instead, overexpression of circRNA HRCR in hypertrophy-induced mice attenuates hypertrophy and interstitial fibrosis by decreasing the expression of stress genes (i.e., β-MHC). This is mainly due to the sponging activity of HRCR on miR-223, a miRNA involved in the induction of hypertrophy after cardiac stress through the inhibition of the ARC (Apoptosis inhibitor with CARD domain target) protein [167]. Recent evidence from mouse studies [168] further supports the therapeutic potential of RNA-based sponges to regulate cardiac hypertrophy. Specifically, Lavenniah and colleagues designed synthetic circ-miR sponges against miR-132 and miR-212, both involved in pathological hypertrophy [104]. Consistently, their intraperitoneal injection produced hypertrophy attenuation and ameliorated cardiac functions in mice subjected to transverse aortic constriction [168].

##### Nuclear ncRNAs

The ability to act as sponges is mostly executed by cytoplasmic RNAs. Nuclear and chromatin enriched lncRNAs act as epigenetic rheostats of myogenesis through a variety of mechanisms [62,65]. Among the most recent examples, LncMAAT (Muscle-Atrophy-Associated Transcript) is a lncRNA that inhibits miR-29b transcription by impeding the binding of the transcription factor SOX6 to its promoter (Figure 3B). LncMAAT overexpression has been proposed as a possible strategy to treat muscle atrophy due to the significant attenuation of the pathological phenotypes (i.e. decreased weight of gastrocnemius muscles and grip strength and increased apoptosis) observed in AngII-induced atrophy mice [169]. Another example of a nuclear regulator is CPR (cardiomyocyte proliferation regulator), a lncRNA acting as a guide for the inhibitory DNMT3A (DNA methyltransferase 3 alpha) factor on the promoter of MCM3 (Minichromosome Maintenance Complex Component 3), whose expression is essential for genome replication and cell cycle progression (Figure 3C) [171,185]. In CPR knock-out mice, cardiomyocytes appear smaller than wild-type ones, although they are equipped with higher renewal capability. Indeed, upon MI, these mice show a higher percentage of proliferating cardiomyocytes accompanied by a clear improvement in cardiac functions, as compared to control animals [171]. Contrarily, the lncRNA Linc-YY1 acts as a decoy for YY1 (Yin-Yang 1) by blocking its interaction with the PRC2 complex, leading to the deregulation of several pro-differentiation genes (Figure 3B) [186]. Depletion of Linc-YY1 by siRNAs in satellite cells caused a significant decrease of MyoG and Pax7 positive cells. This result was also mirrored in vivo in cardiotoxin-induced mice in addition to a reduced number of newly formed myofibers [170]. A further example is Lnc-Rewind (Repressor of wnt induction), a chromatin-associated lncRNA previously identified by transcriptomic analysis [187] and recently shown to act as an epigenetic regulator of satellite cells proliferation and expansion [172]. Mechanistically, Lnc-Rewind directly interacts with the methyltransferase G9a to mediate the repression of its neighboring gene, Wnt7b, the expression of which is important for satellite cells’ differentiation (Figure 3C). Consistently, the depletion of Lnc-Rewind in MuSC-derived myoblasts leads to Wnt7b de-repression and to the aberrant expansion and activation of satellite pools on single myofibers [172]. Finally, we cannot fail to mention H19, one of the earliest lncRNAs to be identified and shown to exert diverse functions in varying types of cells, including satellite, cardiac, and skeletal muscle cells [188,189]. H19 represents an optimal candidate to target multiple tissues at the same time. Indeed, by generating a mouse model with a specific mutation on the dystrophin gene (C3333Y), Zhang and colleagues were able to demonstrate that the overexpression of H19 through the subcutaneous injection of an H19-conjugated agrin peptide (AGR-H19) leads to increased dystrophin levels in both cardiac and skeletal muscle. This treatment not only resulted in increased skeletal muscle strength and a reduced percentage of immature fibers but also amelioration of cardiac functional parameters [190]. Notably, cardiomyocytes-specific overexpression of H19 in mice by AAV9 delivery was also shown to decrease heart weight and cardiomyocytes size in both physiological and hypertrophy-induced conditions [191]. Even though the molecular mechanism is still not clear, H19 knock-out mice display a 50% decrease in the satellite cell pool and an abnormal skeletal muscle regeneration capability after injury, suggesting its importance in the maintenance of quiescent satellite cell pools [188]. Our citations likely exclude other valuable examples of cytoplasmic and nuclear lncRNA-related circuitries. However, these paradigmatic examples already point out the important role that lncRNAs hold in influencing the regeneration potential of skeletal and cardiac muscle.

## 4. RNA as a Diagnostic Molecule for Muscle Diseases

Together with clinical treatment, the possibility to identify a pathological condition quickly and precociously is extremely important to prevent the worst outcomes. For this reason, studies aimed at the identification of specific RNA biomarkers for different diseases have been steadily growing in the latest years. Both coding and ncRNAs have been found in nearly all peripheral bodily fluids [192,193] and could help fill the void of reliable biomarkers.

In muscular diseases, the measurement of circulating biomarkers can lead to extremely rapid, non-invasive, and easy-to-perform diagnostic paths, which overcome the need for surgical biopsies. An increasing number of studies have demonstrated the validity of using circulating miRNAs as biomarkers for muscular disorders, such as DMD and DM1. For instance, the expression of miR-1, miR-206, and miR-133 myo-miRs was found to be high in the serum of DMD patients and strongly correlated to disease severity [194,195]. However, as their expression decline with age [196], probably due to the progressive loss of skeletal muscle mass, it is extremely hard to use them as markers in patients. Indeed, it is difficult to discriminate whether their levels are reduced during the pathology due to treatment or age. Another miRNA, miR-483-5p, has been added as a potential biomarker for DMD. Even though it has a lower predictive power in respect to myo-miRs, miR-483-5p expression levels are unchanged with age, thus offering an advantage in monitoring the progress of treated patients [197]. Together with myo-miRs, the pool that includes miR-27b, miR-140-3p, miR-454 and miR-574 can significantly discriminate DM1 patients from healthy controls if analyzed in combination or alone [80]. Their abundance in plasma correlates well with skeletal muscle strength and the levels of creatine kinase, which confirm the potential of miRNAs as biomarkers.

Identifying new biomarkers is especially important for cardiac diseases, in consideration of the often-abrupt way they occur. In 2016, Vausort and colleagues purified circMICRA from the plasma of more than 600 acute MI patients and demonstrated its possible use as a biomarker for left ventricular dysfunction [198]. CircYOD-1 was instead found as a biomarker for coronary heart disease together with two miRNAs (hsa-miR-21-3p and hsa-miR-296-3p) and thirteen mRNAs [199]. Clinical trials are also currently ongoing for the early detection of cardiac complications. For instance, a new test based on the blood levels of nine mRNAs (HEARTBiT) has been successful in predicting heart transplant rejection in a pilot study of 16 patients [200]. HEARTBiT is currently being assessed in a bigger sample size (NCT03575910) (Table 1). CRUCIAL (Circulating RNAs in acute congestive heart failure) instead aims to examine circulating RNAs in patients with acute congestive heart failure, to find candidates able to predict structural changes and increased fibrosis post-MI (NCT03345446) (Table 1). Finally, one of the challenges presented by MI recovery is the tendency to develop left ventricular dysfunction and remodeling characterized by ventricular enlargement and hypertrophy [201]. In this context, the treatment with β-blockers, initially used for the prevention of secondary MI events by hormone (adrenaline or noradrenaline) inhibition [202], has also shown to be effective in the prevention of cardiac remodeling. The BORG clinical study [203] was undertaken with the purpose to understand the gene expression changes caused by treatments with β-blockers and eventually predict pathological cardiac remodeling. After 3 months of treatment with β-blockade drugs, a decreased expression of miR-208a-3p and miR-591 was identified and used to predict time-dependent heart remodeling in 43 patients with idiopathic dilated cardiomyopathy. After 12 months of treatment, altered expression of four more miRNAs (miR-208b-3p, miR-21-5p, miR-199a-5p, and miR-1-3p) was identified, with all of them being downregulated in patients subjected to β-blockade treatment, except for miR-1-3p [203].

## 5. Conclusions and Perspectives

RNA shows incredible potential for both diagnosis and treatment of a vast number of diseases, including muscular and cardiovascular pathologies. The use of RNA-based drugs has several advantages, mainly (i) the quick and easy method of design, (ii) the high specificity in target recognition, mostly achieved by base-pairing, (iii) the possibility to target specific cell types or tissues, and (iv) their functional versatility. The usefulness and reliability of small ncRNAs-based drugs (i.e., miRNA, ASO, and siRNA) have already been recognized by the competent FDA and EMA institutions, which have given approval for their use in SMA and DMD. Long RNAs also represent appealing candidates for the development of innovative approaches. Chemical modifications to improve mRNA stability and prevent its immunogenicity have also allowed researchers to pursue their use for the treatment of several conditions. As of now, clinical trials that are being conducted to test the effect of modRNAs expression are still in Phase I or II; however, they already show promising results for future use in human patients. Among the non-coding species, lncRNA-based drugs could be exploited to directly target the nucleus, thus influencing the early stages of gene expression, such as gene transcription, epigenetic regulation, and RNA processing. Despite several studies demonstrating the feasibility of using these molecules for therapeutic purposes in animal models, their application in human patients is still far from being tested. Nevertheless, it is undeniable their potential to revolutionize, in the future, the approaches to therapeutic treatments.

## Figures and Tables

**Table 1:** RNA-based drugs and biomarkers for cardiac and skeletal pathologies.

Drug	RNA Type	Target	Disease/Condition	Company	Phase	Reference
MRG-110	Anti-miR	miR-92a	Wound Healing	miRagen (Viridian)	Phase I	NCT03603431
Spinraza (Nusinersen)	ASO	SMN2	SMA	Ionis	FDA/EMA approved	NDA:209531 EMEA/H/C/004312
Eteplirsen (Exondys 51)	ASO	Dystrophin	DMD	Sarepta Inotersen	FDA approved	NDA:206488
Drisapersen (Kyndrisa)	ASO	Dystrophin	DMD	BioMarin	Phase III	NCT02636686
Vitolarsen (Viltepso)	ASO	Dystrophin	DMD	Nippon Shinyaku	FDA approved	NDA:212154
Golodirsen (Vyondis 53)	ASO	Dystrophin	DMD	Sarepta Therapeutics	FDA approved	NDA:211970
Casimersen (Amondys 45)	ASO	Dystrophin	DMD	Sarepta Therapeutics	FDA approved	NDA:213026
TQJ230	siRNA	Apo(a)	Cardiovascular Disease, Elevated Lp(a)	Novartis	Phase III	NCT04023552
AZD8601	mRNA	VEGF	Ischemic Heart Disease	Moderna, Astrazeneca	Phase II	NCT03370887
HEARTBiT	miR	Biomarker	Heart Transplant Rejection			NCT03575910
CRUCIAL	Circulating RNAs	Biomarker	Acute Heart Failure			NCT03345446

## Data Availability

Not applicable.
